# Overcoming the challenges in translational development of natural killer cell therapeutics: An opinion paper

**DOI:** 10.3389/fonc.2022.1062765

**Published:** 2022-12-02

**Authors:** Hong Qin, Changqiao You, Feng Yan, Kefang Tan, Changgen Xu, Rui Zhao, Marlene Davis Ekpo, Songwen Tan

**Affiliations:** ^1^ Research and Development Department, NanHua Bio-medicine CO., Ltd., Changsha, China; ^2^ Xiangya School of Pharmaceutical Sciences, Central South University, Changsha, China

**Keywords:** natural killer (NK) cells, immunosenescence, immunotherapy, cancer, chemotherapy, biotherapeutics

## Introduction

Cellular therapies have attracted huge research and clinical attention lately (1, 2). Natural killer cells (NKCs) are a class of innate immune lymphoid cells (ILP) mainly derived from bone marrow lymphoid stem cells (3). They are mainly distributed in peripheral blood (PB) and peripheral lymphoid tissues, accounting for about 10% of the total lymphocytes in PB (4–7). Presently, NK cell immunotherapeutics utilize cells derived from many sources including PB, umbilical cord blood (8), immortalized NK cell lines, and more recently, induced pluripotent stem cells (iPSC) (9–11). They are characterized by rapid response and non-specific cytotoxic effect without prior antigen sensitization, and are independent on antibodies or complements (12, 13). NKCs induce apoptosis of target cells by secreting perforin, granzyme, cytokines and chemokines (14, 15). NKCs also selectively attacks foreign and diseased cells through expression of killer-cell immunoglobulin-like receptors (KIRs) and Fc receptor (CD16) where the later mediates antibody-dependent cell-medicated cytotoxicity (ADCC) (16–18).

Human NKCs are defined by the CD3^-^CD56^+^ surface phenotype (19, 20). According to their surface expression of CD56, they are divided into two main subpopulations: CD56bright and CD56dim, which differ significantly in biological characteristics (20–22). Briefly, CD56bright NKCs are immature cells and the progenitor for effector cells with high expression of CD56 and low expression of CD16 and KIRs (21), and accounts for about 5- 10% of the total NKCs. Also, they are weakly immunoregulatory and rely mainly on the secretion of cytokines, growth factors and chemokines (22). On the contrary, CD56dim subset accounts for about 90-95% of the circulating NKCs. They are characterized by low expression of CD56 and high expression of CD16, KIRs, FcγRIII and a variety of NK cell inhibitory receptors. They exhibit intrinsic cytotoxicity and ADCC but have weak cytokine secretion ability (23, 24).

Many studies have revealed the role of NKCs in pathologies like autoimmune diseases (25, 26), leukemia (25), pregnancy-related conditions (27), liver diseases (28–30), HIV (31), HPV (32), atherosclerosis (33) and in many age-related diseases. Aging is usually associated with increased susceptibility to infectious diseases and cancers due to immunosenescence which is particularly reflected in the biological changes in the population and subsets of NKCs throughout life (34). As the functioning of the immune system decreases with age, NKCs have difficulties in initiating adaptive immune responses and mobilizing effective immune molecules, leading to the occurrence of diseases related to aging, such as infections and tumors (35–38). The proportion of CD56dim NKCs in the elderly increased with age, while the CD56bright NKCs decreased significantly, suggesting that the increased CD56dimNK : CD56brightNK ratio is significantly age related (39, 40). Studies have also shown that the mortality risk in the elderly with low NK cell counts is three-fold higher than that in the elderly with high NK cell counts (41). In addition to the decreased CD56bright NKCs in older adults (42–44), Sagiv et al. demonstrated that the decline in perforin-mediated NK cytotoxicity is similarly age-related, and may hinder the ability of NKCs to clear senescent cells in the elderly (45). Similarly, NKCs from geriatric population have synonymous reduction in proliferative response to interleukin 2 (IL-2) and expression of the CD69 activation antigen (46–49). With immunosenescence being almost inevitable, many are willing to explore therapies to escape the negative consequences of aging especially tumor development which is increasingly prevalent. Therefore, undergoing NK cell therapy in early old age may help particularly in alleviating cancers.

Studies have shown that the therapeutic efficiency of NK cell therapeutics while encouraging in hematopoietic malignancies, is unsatisfactory in solid tumors as it is problematic for NKCs to infiltrate tumor sites (50, 51). The function, activation, and persistence of NKCs are significantly diminished by the tumor microenvironment (TME), leading to their dysfunction or exhaustion. In this paper, we wish to draw the attention of researchers to the fact that NK cellular products are highly promising in the fight against cancer and other age-complicated diseases and if they must be applied safely, more efforts should be directed towards addressing the bottlenecks. Broadly, NK cell infiltration, solid tumor targeting, *in vivo* persistence and resistance to TME must be improved, and reproducible and standardized protocols must be developed for the generation and expansion of NKCs. Here in, we highlight the strategies employed in tackling the challenges as this will serve as guide to the research trend and future directions considered in the development of clinical grade NK biotherapeutics.

## Improving tumor targeting, microenvironment resistance and solid tumor infiltration

Clinical trials involving NKCs is generally not a new topic as they have been in progress for over two decades from where preliminary data regarding the safety and efficacy of NKCs have been obtained following their adoptive transfer to treat hematologic malignancies (25, 52). Nevertheless, some limitations have been encountered in the application of NK cell therapy to solid tumors largely because the TME harbors suppressive ligands, metabolites and cytokines which threatens the survival of NKCs (53). Additionally, the tumor itself possesses other defense mechanisms against attack by NKCs. Therefore, enormous research efforts are directed towards producing or modifying NKCs to be more resistant to attack from tumors and TME while harnessing their cytolytic effect after tumor penetration (54, 55). One of such approaches include blocking of inhibitory receptors with monoclonal antibodies (mAbs) like monalizumab (56).

Increasing the efficacy of tumor cell recognition is achievable *via* genetic modification (57) (58). For example, CAR-NKCs have enhanced cytolytic activity attributable to the synergistic effect of targeted specificity against tumor associated antigens and intracellular signaling of receptors (59, 60). CAR-NKCs can also be fashioned with receptors for a wide range of antigens with the CAR expression permitting carrier cells to recognize antigens on tumor-cell surfaces without major histocompatibility complex restriction (9, 58, 61). Also, unlike CAR-T cell therapies, CAR-NKCs possess reduced risk of cytokine release syndrome, neurological complications and better potential for allogeneic applications (62).

Transfection efficiency for primary NKCs is a key obstacle to the large-scale manufacture of genetically modified CAR-NKCs and different techniques like viral transduction and non-viral electroporation are underway to addressing this challenge (63, 64). Kumar et al. has recently led the production and evaluation of CRISPR-engineered NK-92 cell constitutively expressing Cas9 or dCas9 which have shown good prospects for further research and possible clinical application (65).

## Pharmacokinetics and pharmacodynamics of natural killer cell therapeutics

NK and other cellular therapeutics are different from conventional (chemical) drugs therefore great disparity exists in their pharmacokinetics and pharmacodynamics properties. There is substantial evidence from clinical studies regarding the safety and efficacy of NK cell therapeutics. Nonetheless, more research is on demand to explore the sensitivity a wider variety of tumors to NK cell therapy, determine the mechanism(s) of action (cytotoxic response) against different types of tumors and identify possible contraindications. Some of the identified issues are as thus:

1. Allogenic NKCs from PB are relatively safe and satisfactorily effective against tumors but are susceptible to rejection by the host (66, 67).
2. Although several studies show the relationship between high NKCs, their receptors/ligands levels and better overall survival in patients with hepatocellular carcinoma (HCC), the underlying mechanism of remains unclear (13).
3. In obese patients, significant numbers of NK and T cells are recruited to the visceral adipose tissue at the expense of successful tumor infiltration and eradication (68), thus posing a serious challenge for the application of NK cell therapy in certain comorbid situations.
4. Combination therapy with NK and T cells or other tumor therapy strategies need to be confirmed with large-scale clinical trials as the clinical outcome can vary between tumor types.

Hence, several research efforts are geared towards unravelling possible NK mechanism of cytolysis like mitochondrial apoptosis (64) and release of perforin and granzymes (69); factors that may increase their cytotoxicity such as E26 transformation-specific transcription factor ELK3 expression by cancer cells (70); factors that attenuate cytolytic function like increased transforming growth factor-beta 1 (TGF-β1) (32), inhibition of O-GlcNAcylation (71), low surface expression and impaired function of transient receptor potential melastatin 3 (TRPM3) (32, 71–75); and addressing the complications accompanying rejection-prone cellular products (67, 76, 77).

## Expansion and activation of natural killer cells

Another obstacle to the manufacture of clinical grade NK cell therapeutics is the large-scale expansion of NKCs without loss of their cytotoxic activity (78). The expansion of NKCs can be *ex vivo* or *in vivo* followed by isolation by CD3+ cell depletion and subsequent positive selection of CD56+ cells. Other strategies involve a single step depletion of CD3+ and CD19+ cells using magnetic beads (79), and differentiation of functional NKCs from enriched CD34+ progenitors present in cord blood and bone marrow (80, 81). While good manufacturing practice (GMP) guidelines have been established, inconsistencies exist between the cytotoxicity, expansion rate, receptor expression, cytokine secretion and phenotype based on their respective source and expansion method (80, 82–84) which may influence their therapeutic activity. iPSCs derived NKCs possess improved expansion rate, cytokine secretion and cytotoxic compared to those from PB. They can also be genetically functionalized to harness tumor targeting, cytolytic activity and persistence in the TME (85). They have been clinically tested for different diseases including graft versus host disease, Parkinson’s disease and heart failure (86, 87). Synergistic activity of iPSCs-derived NKCs with other effector T-cells and successful *in vitro* tumor infiltration has been described in animal studies (86). Other studies in this regard are focused on developing efficient methods and designing biomaterials for activation, expansion and isolation of NKCs (85, 88–91). Gao et al. recently classified the biological and transcriptomic signatures of cord blood and placenta-derived NKCs revealing the cellular/molecular level similarities and differences existing between the NK cell types (92). More of such studies are needed as they serve as database for cell-based immunotherapy and will be beneficial to understanding and categorizing the mechanism of action of different NKCs.

## Optimizing persistence and cytolytic activity of natural killer cells

There is a huge need of novel technologies to enhance the activity of NKCs and their interaction with tumors. Consequently, several methods have been proposed. For instance, concomitant use of NK cell adoptive transfer and other therapeutic methods, including T-cells, chemotherapeutic agents, cytokines and immunomodulatory drugs could fortify NKCs against the TME and be synergistic in tumor immunotherapy (93–95). The biological targets of these supplementary molecules like cytokines and drugs vary from those of NKCs providing synergy (96) but their safety must also be assured before clinical application. Biber et al. describes the design of a non-viral lipid nanoparticle-based delivery system that encapsulates small interfering RNAs which targets NKCs *in vivo*, silences inhibitory molecules, and activate NK cell anti-tumor activity (97). Park et al. reports that *Aurantii Fructus Immaturus*, a commonly used herb in traditional medicine enhances the anticancer efficacy of NK (98), Bispecific killer cells engagers (BiKEs) and trispecific killer cells engagers (TriKEs) improve *in vitro* secretion of cytokines and efficiently induce the cytotoxic effects of NKCs (99, 100). Moving forwards, optimizing and improving these formulations to avoid undesirable side effects are vital steps toward their clinical application.

## Conclusion

The role of NKCs in neutralizing senescent, stressed and malignant cells has attracted enormous research attention aimed at producing clinical grade NKCs for adoptive cell immunotherapy. Currently, some clinical studies are designed to determine the safety and efficacy of ex vivo activated and expanded NKCs while others test the effect of administering the NKCs in combination with other immune molecules. Major advances, including the development of efficient *ex vivo* expansion systems, prolonged *in vivo* persistence and genetic manipulation strategies involving CARs are currently explored to facilitate clinically applicable NK cell therapeutics. However, additional research effort is needed to enhance tumor targeting, overcome immune suppression by inhibitory signals or cells and exhaustion in the TME, increase persistence in allogeneic settings, facilitate expansion in patients, sustain *in vivo* surveillance against tumor relapse, and increase the applicability of NK cell therapy to a wider range of life-threatening diseases especially those marked by depletion in NK cell function. Finally, iPSC-NKCs hold great prospects and further refinement of their differentiation protocol is necessary to match the phenotypic properties of PB NKCs.

## Author contributions

Conceptualization, HQ, CY, FY, KT, CX, and ME. Writing—original draft preparation, HQ, ME, RZ, and ST. Writing—review and editing, FY, ME, KT, CX, and ST. Supervision and approval, HQ, CY, and ST. All authors contributed to the article and approved the submitted version.

## Conflict of interest

Authors HQ, CY, FY, KT, CX were employed by company NanHua Bio-medicine CO., Ltd.

The remaining authors declare that the research was conducted in the absence of any commercial or financial relationships that could be construed as a potential conflict of interest.

## Publisher’s note

All claims expressed in this article are solely those of the authors and do not necessarily represent those of their affiliated organizations, or those of the publisher, the editors and the reviewers. Any product that may be evaluated in this article, or claim that may be made by its manufacturer, is not guaranteed or endorsed by the publisher.
